# Percutaneous Mitral Valve Repair: Outcome Improvement with Operator Experience and a Second-Generation Device

**DOI:** 10.3390/jcm10040734

**Published:** 2021-02-12

**Authors:** Xavier Freixa, Rodrigo Estévez-Loureiro, Fernando Carrasco-Chinchilla, Xavier Millán, Ignacio Amat-Santos, Ander Regueiro, Luis Nombela-Franco, Isaac Pascual, Belen Cid, José Ramón López-Mínguez, Rosa Ana Hernández-Antolín, Ignacio Cruz-González, Leire Andraka, Javier Goicolea, Valeriano Ruíz-Quevedo, Jose Luís Díez, Alberto Berenguer, José Antonio Baz, Manuel Pan, Tomas Benito-González, Juan H. Alonso Briales, Chi Hion Li, Laura Sanchis, Ana Serrador, Pilar Jiménez-Quevedo, Pablo Avanzas, Luisa Salido, Felipe Fernández-Vázquez, José Maria Hernández-García, Dabit Arzamendi

**Affiliations:** 1 Hospital Clinic de Barcelona, Institut Clínic Cardiovascular, 08036 Barcelona, Spain; xavierfreixa@hotmail.com (X.F.); anderregueiro@gmail.com (A.R.); lsanchis@clinic.cat (L.S.); 2 Hospital Universitario de León, 24071 León, Spain; roiestevez@hotmail.com (R.E.-L.); tomasbenito@outlook.com (T.B.-G.); ffernandez@secardiologia.es (F.F.-V.); 3 Hospital Universitario Virgen de la Victoria, 29010 Málaga, Spain; fernandocarrascochinchilla@gmail.com (F.C.-C.); juanhalonso62@gmail.com (J.H.A.B.); josemaria2509@gmail.com (J.M.H.-G.); 4 Hospital de la Santa Creu i Sant Pau, 08041 Barcelona, Spain; xmillanalvarez@gmail.com (X.M.); ch.pedroli@gmail.com (C.H.L.); dabitarza@gmail.com (D.A.); 5 Hospital Clínico Universitario de Valladolid, 47003 Valladolid, Spain; ijamat@gmail.com (I.A.-S.); aserradorf@gmail.com (A.S.); 6 Hospital Clínico de San Carlos, 28040 Madrid, Spain; luisnombela@yahoo.com (L.N.-F.); patropjq@gmail.com (P.J.-Q.); 7 Hospital Central de Asturias, 33011 Oviedo, Spain; avanzas@gmail.com; 8 Complejo Hospitalario Universitario de Santiago, 15706 Santiago de Compostela, Spain; belcid77@hotmail.com; 9 Hospital Universitario Infanta Cristina, 06080 Badajoz, Spain; lopez-minguez@hotmail.com; 10 Hospital Ramón y Cajal, 28034 Madrid, Spain; rhernandez_antolin@hotmail.com (R.A.H.-A.); luisasalido@hotmail.com (L.S.); 11 Hospital Clínico Universitario de Salamanca, 37007 Salamanca, Spain; cruzgonzalez.ignacio@gmail.com; 12 Hospital Civil de Basurto, 48013 Basurto, Spain; Leire.andrakaikazuriaga@osakidetza.eus; 13 Hospital Puerta de Hierro, 28222 Madrid, Spain; j_goicolea@hotmail.com; 14 Complejo Hospitalario de Navarra, 31008 Pamplona, Spain; valeriano.ruiz.quevedo@navarra.es; 15 Hospital La Fe de Valencia, 46026 Valencia, Spain; diez_jlu@gva.es; 16 Hospital General Universitario de Valencia, 46014 Valencia, Spain; berenguer_alb@gva.es; 17 Complejo Hospitalario Universidad de Vigo, 36310 Vigo, Spain; joseantoniobaz@gmail.com; 18 Hospital Reina Sofía de Córdoba, 14004 Córdoba, Spain; manuelpanalvarez@gmail.com

**Keywords:** transcatheter mitral valve repair, mitral regurgitation, MitraClip

## Abstract

Background and aim: Recent randomized data comparing percutaneous mitral valve repair (PMVR) versus optimal medical treatment in patients with functional MR (FMR) seemed to highlight the importance of the learning curve not only for procedural outcomes but also for patient selection. The aim of the study was to compare a contemporary series of patients undergoing PMVR using a second-generation Mitraclip device (Mitraclip NT) with previous cohorts treated with a first-generation system. Methods: This multicenter study collected individual data from 18 centers between 2012 and 2017. The cohort was divided into three groups according to the use of the first-generation Mitraclip during the first (control-1) or second half (control-2) or the Mitraclip NT system. Results: A total of 545 consecutive patients were included in the study. Among all, 182 (33.3%), 183 (33.3%), and 180 (33.3%) patients underwent mitral repair in the control-1, control-2, and NT cohorts, respectively. Procedural success was achieved in 93.3% of patients without differences between groups. Major adverse events did not statistically differ among groups, but there was a higher rate of pericardial effusion in the control-1 group (4.3%, 0.6%, and 2.6%, respectively; *p* = 0.025). The composite endpoint of death, surgery, and admission for congestive heart failure (CHF) at 12 months was lower in the NT group (23.5% in control-1, 22.5% in control-2, and 8.3% in the NT group; *p* = 0.032). Conclusions: The present paper shows that contemporary clinical outcomes of patients undergoing PMVR with the Mitraclip system have improved over time.

## 1. Introduction

Percutaneous mitral valve repair (PMVR) with the Mitraclip system has been growing over the last years. With more than 200,000 implants worldwide, the therapy seems to be settling down as one of the available tools for mitral regurgitation (MR) treatment in several reference centers. Initial data from the randomized EVEREST II trial [1,2] and other international registries showed consistent and promising results with the use of Mitraclip for functional (FMR) and degenerative MR (DMR) patients [3,4,5,6,7]. However, recent data from the MITRA-FR [8] and COAPT trials [9], two randomized trials comparing Mitraclip versus optimal medical treatment in FMR patients, showed opposite results. Whereas MITRA-FR showed no clinical benefit with the use of Mitraclip, COAPT showed a significant reduction in mortality and congestive heart failure (CHF) admissions. In fact, clinical improvement after PMVR is related to several factors such as the clinical status of the patient before the intervention, the presence of comorbidities, or the MR degree before and after the procedure [10,11]. All these factors, somehow linked to the overall learning curve of the procedure, seemed to differ between these two randomized trials and might explain the differences in clinical outcomes.

PMVR with Mitraclip is a demanding procedure that requires advanced interventional and imaging skills. The device has experienced some technical improvements intended to ease its use, improve MR reduction, and reduce the number of complications [12]. In addition, patient selection seems to be moving to those that can present a higher benefit from MR correction such as those with a non-terminal cardiac condition [13,14]. For all these reasons, overall outcomes after Mitraclip implantation seem to be improving during the last years. The present paper aims to compare a contemporary series of patients undergoing Mitraclip with a second-generation device (NT system) with a previous cohort treated with first-generation Clip (MitraClip1G). The present manuscript tries to explore if the implemented design improvements in the NT system along with the growing experience and patient selection have had a relevant impact on clinical and echocardiographic outcomes.

## 2. Materials and Methods

### 2.1. Study Population

This multicenter study collected individual data from the Spanish Mitraclip Registry. Patients who underwent PMVR with Mitraclip between 2012 and 2017 from 18 centers in Spain were included in the registry. For the purpose of our study, the cohort was divided according to the use of the first-generation Mitraclip (control group) or the Mitraclip NT system (NT cohort). In addition, in order to evaluate the specific effects of the learning curve, the control group (first-generation Mitraclip) was divided into two halves on the basis of the date of implantation (control-1 and control-2).

The indication for MitraClip was agreed upon by a multidisciplinary team comprising cardiologists, cardiac imaging experts, cardiac surgeons, and anesthetists. Demographics, baseline and procedural characteristics, left-ventricular ejection fraction LVEF, and clinical/echocardiographic outcomes at follow-up results were prospectively collected in an online dedicated, shared, and prospective database at each participating center. Most of the procedures were performed under general anesthesia and guided by transesophageal echocardiogram (TEE) and fluoroscopy/angiography. Periprocedural complications were noted during the index hospitalization. The study was conducted in accordance with the institutional ethics committee of each participating center, and all patients provided signed informed consent for the procedure. In order to balance follow-up data among groups, all events were analyzed up to 12 months after the index procedure.

### 2.2. Post-Procedural Care

Antithrombotic management was based on an individualized protocol. Patients receiving warfarin or direct oral anticoagulants prior to clip placement continued on an identical warfarin regimen after the intervention. In the remainder of patients, single or dual antiplatelet treatment was prescribed according to local consensus; however, in general, acetylsalicylic acid (75–150 mg/day) was prescribed for 3–6 months and clopidogrel (75 mg/day) was prescribed for 4 weeks. Clinical and echocardiographic follow-up was performed at discharge, 3–6 months and 12 months post procedure.

Echocardiographic data at follow-up were reported by participating centers. Mitral regurgitation was graded according to the American Society of Echocardiography guidelines [1].

### 2.3. Endpoints and Definitions

We compared the study groups using the following endpoints: (1) primary composite endpoint: freedom from death, MR surgery after clip and hospital admission for CHF within the first year after the procedure; (2) procedural success: MR reduction to grade ≤2+ (moderate) after clip implantation [1,2]; (3) procedural time: time from anesthesia induction to the end of the procedure; (4) device time: time from the insertion of the delivery system to clip delivery system removal.

### 2.4. Statistical Analysis

Results are presented as the mean ± standard deviation for continuous normally distributed variables, as the median (interquartile range) for continuous non-normally distributed data, and as percentages for categorical data. Analysis of normality was performed with the Kolmogorov–Smirnov and Shapiro–Wilk tests. Categorical data and proportions were compared using a *χ^2^* test or Fisher’s exact test as required. Comparisons of continuous variables were analyzed using an unpaired *t*-test and the Mann–Whitney U-test as appropriate. Survival analysis and survival curves were calculated using the Kaplan–Meier method, and a comparison was obtained with the log-rank test. Multivariable analysis was estimated using the Cox proportional hazards regression model. Considering the variable follow-up among patients, survival curves were constructed at 1 year follow-up as it was a meaningful timeline value, as well as had a sufficient number of patients to perform a valid analysis.

A *p*-value less than 0.05 was considered significant. All data were analyzed with R statistical software, version 3.3.2 (R Foundation for Statistical Computing, Vienna, Austria).

## 3. Results

A total of 545 consecutive patients with MR who underwent PMVR with Mitraclip were included in the study. Among all, 182 (33.3%), 183 (33.3%), and 180 (33.3%) underwent mitral repair with the first-generation Mitraclip (control-1 and control-2 groups) and Mitraclip NT (NT cohort), respectively. No significant baseline clinical and echocardiographic differences were observed between both groups, but a higher proportion of females, older patients, atrial fibrillation, DMR, and higher Society of Thoracic Surgeons. (STS) score was identified in the NT group (Table 1).

Procedural characteristics are shown in Table 2. Patients in the NT group presented shorter device and procedural times, as well as a lower number of implanted clips. In addition, immediate residual MR after Mitraclip implantation was lower in the NT group. Procedural success was achieved in 93.3% of patients without differences between groups. Post-procedural in-hospital clinical outcomes and major adverse events did not statistically differ among groups, but there was a higher rate of pericardial effusion in the control-1 group (Table 3). Patients in the NT group presenter shorter hospital admissions as compared to those in the control group.

### 3.1. Clinical Follow-Up

**Figure 1:**
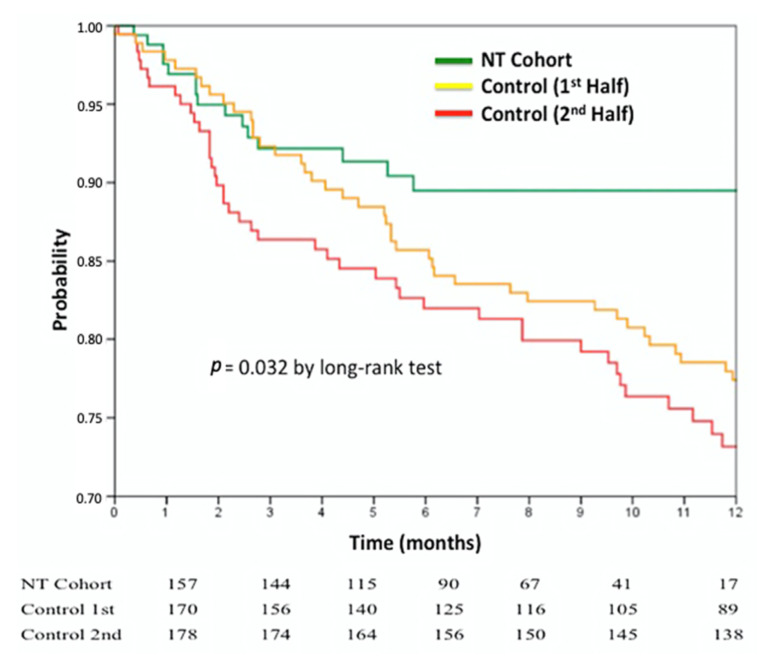
Freedom from death, surgery, and CHF admission.

After a median follow-up of 12 (6.9–12) and 6 (2.3–9.7) months in the control and NT group, respectively, 87% of the patients were alive (86.0% in control-1, 77.5% in control-2, and 96.7% in the NT group; *p* = 0.167). None of the patients in the NT group underwent mitral surgery at follow-up (1.6% in control-1, 1.6% in control-2, and 0% in the NT group; *p* = 0.436). The reason for mitral valve surgery at follow-up was the presence of a residual severe mitral regurgitation in all cases. Overall, 12.1% of patients required at least one hospital admission for CHF at 12 months. Importantly, patients in the NT group presented a trend toward a lower need for admissions than those in the control group (14.8% in control-1, 16.5% in control-2, and 5% in the NT group; *p* = 0.061). The composite endpoint of death, surgery, and admission for CHF was lower in the NT group (23.5% in control-1, 22.5% in control-2, and 8.3% in the NT group; *p* = 0.032) (Figure 1). When analyzing FMR and DMR patients separately, only those with FMR presented a trend toward significant differences in the composite endpoint among both control groups and the NT cohort.

In a multivariable analysis including the three clip groups, age, gender, MR etiology, atrial fibrillation, and STS score, the only group with higher death, surgery, or hospital admission for CHF at 12 months was the second half of the control group (hazard ratio (HR) 2.036; 95% confidence interval (CI) 1.013–4.091; *p* = 0.046).

**Figure 2:**
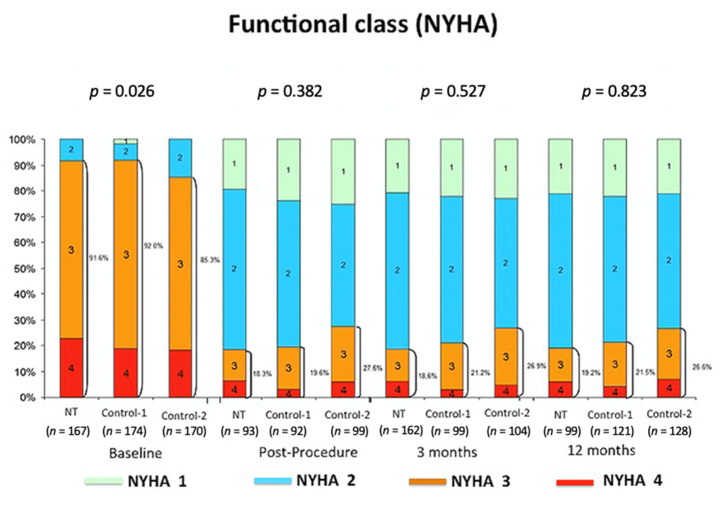
Functional class New York Heart Association (NYHA) at follow-up.

From the clinical perspective, there was a relevant clinical improvement before and after the intervention (Figure 2). At 12 months follow-up, 78.5%, 73.4%, and 80.8% of the patients in control-1, control-2, and NT group, respectively, presented functional class 1 or 2 (New York Heart Association, NYHA) without significant differences between groups.

### 3.2. Echocardiographic Follow-Up

**Figure 3:**
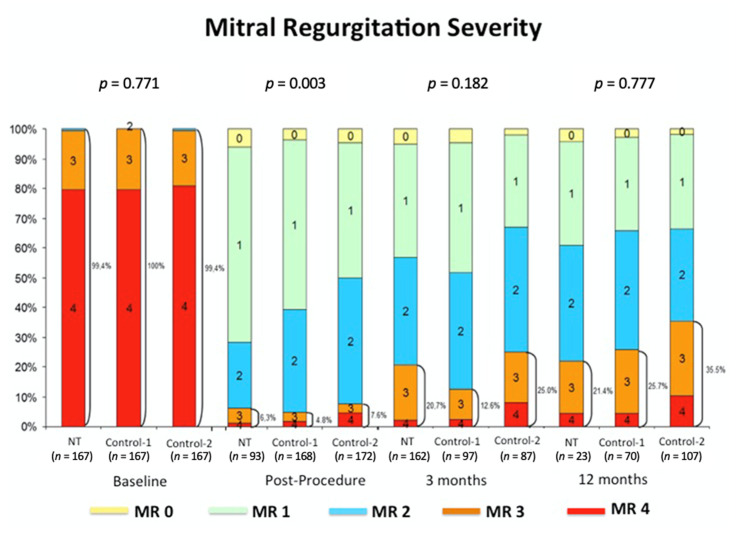
MR severity at follow-up.

As shown in Figure 3, there was a significant reduction in the degree of MR before and after the implantation of Mitraclip. Nonetheless, despite a constant numerical difference in favor of a lower degree of MR in the NT group, no significant statistical differences at 3 and 12 months were observed. At 12 months, residual MR ≥3+ was present in 21.4% of patients in the NT group, 25.7% of patients in the control-1 group, and 35.5% of patients in the control-1 group.

## 4. Discussion

The main findings of the present study were as follows: (1) patients in the NT group presented a lower rate of the composite endpoint of death, surgery, and admission for CHF at 1 year; (2) procedural success was high in all groups without significant differences between them; (3) no differences in procedural MAEs were observed among groups; (4) residual MR after the procedure was lower in the NT cohort although this difference was not seen at 12 months.

Overall results for the main clinical outcomes are in agreement with previous published series and registries [3,4,5,6,7]. In the European Sentinel Registry [6], procedural success was achieved in 95% of cases, whereas in-hospital and 1 year mortality were 2.9% and 15.3%, respectively. At 1 year, 22.8% of patients had at least one admission for CHF and 6% of patients had severe (4+) residual MR. The Italian registry (GRASP) [3] reported a rate of 24.2% for the composite endpoint (death, surgery, and CHF admission) at 12 months with 25% of patients presenting a residual MR ≥3+ by this period. In the German registry (TRAMI) [4], procedural success was achieved in 94% and mortality rate was 20.3% at 1 year. The ACCESS European Union (EU) registry showed a mortality rate at 30 days and 12 months of 3.4% and 18.2%, respectively, and 21.1% of patients presented residual MR ≥3+ at 12 months. In contrast, our clinical outcomes clearly differed with those published in the MITRA-FR, a randomized trial in FMR patients comparing the first-generation Mitraclip and optimal medical treatment [8]. Surprisingly, the mortality (24.3%) and CHF admissions (54.6%) at 1 year almost doubled the reported rates in several international registries. Along with our results, MITRA-FR results probably reflected the pivotal role of patient selection in FMR patients [15]. In contrast to MITRA-FR, COAPT patients were on maximal medical treatment before the procedure, baseline MR severity was higher, LV enlargement was lower, and both acute and 12 month residual MR after Mitraclip were lower All these factors translated into better mortality (29.1%) and CHF admission (35.7%) rates at 24 months compared to optimal medical treatment alone [9].

The present manuscript tries to explore if the implemented design improvements in the NT system along with the growing experience and patient selection had a relevant impact on clinical and echocardiographic outcomes. Interestingly, a recent published registry of 231 patients (79 with the NT system) showed no differences in clinical and echocardiographic outcomes both in hospital and at 12 months follow-up [12]. In contrast, in our series, the NT cohort presented a lower rate of the composite of death, surgery, and admission for CHF at 1 year as compared to the control group. This difference was mainly driven by a reduction in the need for CHF admissions after the procedure and might translate not only the effects of device design improvement but also the learning curve for both procedural and patient selection aspects [11,15,16]. In fact, as shown in Table 1, the main difference in patient selection was the higher rate of DMR in the NT group. Although there were no differences among groups when analyzing only DMR patients, the higher proportion of DMR patients in the NT group may explain the better clinical outcomes in the whole cohort (DMR + FMR). In addition, immediate results after clip implantation showed a lower degree of residual MR with the NT system (Table 2) that, despite not being present at 12 months, may explain the aforementioned observations. Other factors that might reflect the technical design advantages of the NT system, as well as the learning curve, were the lower number of implanted clips and the shorter procedural and device times (Table 2). The specific impact of the learning curve was explored in the control group as patients were divided into two halves according to the date of procedure. As shown in Figure 1, the “learning curve” effect was not seen among the two halves of the control group as depicted by the lack of differences in the primary endpoint. Certainly, the actual impact of both patient selection and procedural learning curve on the study results is very hard to ascertain in a multicenter registry. In any case, regardless of the individual role of every factor (NT design, learning curve, and patient selection), the present paper provides the clinical and echocardiographic outcomes of a contemporary series (NT cohort) compared to a more classic one (control cohort). According to our results, clinical outcomes of patients undergoing mitral repair seem to be improving over the years. In the NT group, the rate of the composite endpoint was 8.3% at 1 year, whereas mortality, surgery, or CHF admissions were 3.3%, 0%, and 5% respectively.

There was a higher prevalence of patients with DMR in the NT cohort (25.9% versus 16.2% in controls). Importantly, the differences in the composite endpoint in favor of the NT group were mainly seen in FMR patients. It is known that patients with FMR and advanced CHF have a worse prognosis despite a successful intervention and this might be the reason why patient selection has changed in order to avoid patients with FMR and advanced cardiomyopathies [11]. In fact, the observed high rates of mortality and CHF admissions at 1 year with FMR in the MITRA-FR trial might somehow reflect the initial experience with the therapy in France [8].

This study had inherent limitations due to it being an observational study without an external adjudication event committee. The results were obtained from an online database prospectively collected in each participating center. No specific information on baseline and post-procedural medical treatment was available. However, this was a post hoc non-prespecified analysis and, even with multivariate analysis, some confounding factors could influence the results. In addition, there is a relevant difference in the number of patients at risk during follow-up among the first-generation and NT cohorts that might have had a potential impact on the final results. Lastly, since the number of cases differed between centers and some centers started the program after 2016, the “learning curve” effect among the control and NT groups might be unbalanced. Additionally, the absence of some specific anatomical or clinical variables regarding the potential differences in the selection of patients might have biased the interpretation of results.

## 5. Conclusions

The present paper shows that clinical outcomes of patients undergoing percutaneous mitral repair with the Mitraclip NT system could be better in comparison with control first-generation cohorts. Further series comparing initial and current experience will be needed to confirm our results.

## Figures and Tables

**Table 1:** Baseline characteristics.

	Total *N* = 545	Control-1 *N* = 182	Control-2 *N* = 183	NT Cohort *N* = 180	*p*
Age	72.7 ± 10.8	70.1 ± 11.5	73.9 ± 9.4	74.2 ± 10.9	<0.001
Gender male (*n* (%))	385 (70.6)	139 (76.4)	131 (71.6)	115 (63.9)	0.031
BMI (kg/m^2^) (*n* = 526/171/355)	27.2 ± 4.7	27.4 ± 5.0	26.6 ± 4.4	27.5 ± 4.5	0.154
BSA (*n* = 526/171/355)	1.81 ± 0.19	1.83 ± 0.21	1.80 ± 0.19	1.80 ± 0.18	0.104
Hypertension	373 (68.4)	118 (64.8)	126 (68.9)	129 (71.7)	0.372
Diabetes mellitus	181 (33.2)	55 (30.2)	63 (34.4)	63 (35.0)	0.570
COPD	119 (21.8)	43 (23.6)	38 (20.8)	38 (21.1)	0.771
Atrial fibrillation	300 (55.0)	88 (48.4)	101 (55.2)	111 (61.7)	0.039
Coronary artery disease	280 (51.4)	29 (15.9)	32 (17.5)	99 (55.0)	0.870
Recent myocardial infarction (<90 days)	29 (5.3)	6 (3.3)	10 (5.5)	13 (7.2)	0.249
Coronary artery bypass graft	89 (16.3)	29 (15.9)	32 (17.5)	28 (15.6)	0.870
Previous percutaneous intervention	189 (34.7)	58 (31.9)	65 (35.5)	66 (36.7)	0.605
STS score	4.3 (2.1–7.5)	3.8 (1.5–6.7)	4.2(2.2–7.5)	4.7 (3.0–8.3)	0.020
LVEF, %	39.1 ± 15.8	36.9 ± 15.3	39.6 ± 16.0	38.3 ± 15.7	0.071
sPAP	52 (41–62)	53 (42–65)	54 (44–61)	50 (40–62)	0.413
LVEDV (mL)	76 (51–151)	87 (58–182)	73 (49–141)	83 (55–155)	0.173
Regurgitant orifice area (mm^2^)	25.3 ± 26.3	28.9 ± 26.3	25.1 ± 26.7	21.5 ± 25.7	0.248
NYHA functional Class					0.026
- I	3 (0.6)	0	3 (1.7)	0	
- II	50 (9.8)	25 (14.7)	11 (6.3)	14 (8.4)	
- III	356 (69.7)	114 (67.1)	127 (73.0)	115 (68.9)	
- IV	102 (20.0)	31 (18.2)	33 (19.0)	38 (22.8)	
MR severity (*n* = 496/162/334)					0.771
- None	0	0	0	0	
- Mild	0	0	0	0	
- Moderate	2 (0.4)	1 (0.6)	0	1 (0.6)	
- Moderate to severe	97 (19.6)	31 (18.6)	34 (20.4)	32 (19.8)	
- Severe	397 (80.0)	135 (80.8)	133 (79.6)	129 (79.6)	
MR etiology					0.003
- Functional	333 (66.5)	126 (75.0)	118 (69.0)	89 (54.9)	
- Degenerative	97 (19.4)	26 (15.5)	29 (17.0)	42 (25.9)	
- Mixed	71 (14.2)	16 (9.5)	24 (14.0)	31 (18.1)	

BMI, body mass index; BSA, body surface area; COPD, chronic obstructive pulmonary disease; CHF, cardiac heart failure; LVEDV Left end diastolic volume; LVEF, left-ventricular ejection fraction; MR, mitral regurgitation; NYHA, New York Heart Association; sPAP, systolic pulmonary pressure; STS, Society of Thoracic Surgeons.

**Table 2:** Procedural characteristics.

	Total *N* = 545	Control-1 *N* = 182	Control-2 *N* = 183	NT Cohort *N* = 180	*p*
Procedural success	454 (93.6)	155 (85.2)	156 (85.2)	143 (93.7)	0.237
Number of clips (mean ± SD)	1.47 ± 0.71	1.65 ± 0.71	1.54 ± 0.66	1.36 ± 0.61	<0.001
Device time (min)	80 (60–100)	100 (60–150)	80 (60–100)	60 (45–92)	<0.001
Procedural time (min)	131 (106–180)	150 (120–240)	120 (100–173)	120 (100–163)	<0.001
MR pre clip					0.221
- None	0	0	0	0	
- Mild	0	0	0	0	
- Moderate	1 (0.2)	1 (0.6)	0	0	
- Moderate to severe	106 (21.2)	28 (16.4)	40 (23.8)	38 (23.5)	
- Severe	394 (78.6)	142 (83.0)	128 (76.2)	124 (76.5)	
MR post clip					0.003
- None	24 (4.8)	8 (4.7)	6 (3.6)	10 (6.3)	
- Mild	278 (55.7)	78 (45.3)	96 (57.1)	104 (65.4)	
- Moderate	166 (33.3)	73 (42.4)	58 (34.5)	35 (22.0)	
- Moderate to severe	18 (3.6)	5 (2.9)	5 (3.0)	8 (5.0)	
- Severe	13 (2.6)	8 (4.7)	3 (1.8)	2 (1.3)	
- Mitral gradient pre-clip	0.61 ± 1.16	0.57 ± 1.33	0.60 ± 1.16	0.71 ± 1.17	0.349
- Mitral gradient post-clip	2.29 ± 1.82	2.27 ± 1.71	2.19 ± 1.86	2.52 ± 1.74	0.056

MR, mitral regurgitation.

**Table 3:** In-hospital outcomes.

	Total *N* = 545	Control-1 *N* = 182	Control-2 *N* = 183	NT Cohort *N* = 180	*p*
Partial or total clip detachment	8 (1.7)	2 (1.2)	3 (2.0)	3 * (1.9)	0.837
Cordal rupture	7 (1.5)	2 (1.2)	2 (1.3)	3 (1.9)	0.857
Cordal entrapment	5 (1.1)	2 (1.2)	2 (1.3)	1 (0.6)	0.810
Femoral pseudoaneurysm	7 (1.5)	3 (1.8)	2 (1.3)	2 (1.3)	0.903
Femoral arteriovenous istula	4 (0.8)	2 (1.2)	1 (0.6)	1 (0.6)	0.819
Transfusion	27 (5.7)	11 (6.7)	10 (6.5)	6 (3.9)	0.501
Vascular surgery	2 (0.4)	1 (0.6)	1 (0.7)	0	0.999
Pericardial effusion	12 (2.5)	7 (4.3)	1 (0.6)	4 (2.6)	0.025
- Medical treatment	8 (1.7)	4 (2.4)	0	4 (2.6)	
- Pericardiocentesis	3 (0.6)	3 (1.8)	0	0	
- Surgery	1 (0.2)	0	1 (0.6)	0	
Air embolism	5 (1.1)	0	2 (1.3)	3 (1.9)	0.112
In-hospital death	11 (2.3)	5 (3.0)	5 (3.1)	1 (0.7)	0.199
Length of stay (days)	4 (2–7)	5 (3–7)	4 (2–6)	3 (2–6)	0.016

* Only one total leaflet clip detachment.

## Data Availability

The data presented in this study are available on request from the corresponding author. The data are not publicly available due to Spanish Mitraclip Registry statement.

## References

[1] Feldman T., Kar S., Elmariah S., Smart S.C., Trento A., Siegel R.J., Apruzzese P., Fail P., Rinaldi M.J., Smalling R.W. (2015). Randomized Comparison of Percutaneous Repair and Surgery for Mitral Regurgitation: 5-Year Results of EVEREST II. J. Am. Coll. Cardiol..

[2] Feldman T., Foster E., Glower D.D., Kar S., Rinaldi M.J., Fail P.S., Smalling R.W., Siegel R., Rose G.A., Engeron E. (2011). Percutaneous repair or surgery for mitral regurgitation. N. Engl. J. Med..

[3] Grasso C., Capodanno D., Scandura S., Cannata S., Immè S., Mangiafico S., Pistritto A., Ministeri M., Barbanti M., Caggegi A. (2013). One- and Twelve-Month Safety and Efficacy Outcomes of Patients Undergoing Edge-to-Edge Percutaneous Mitral Valve Repair (from the GRASP Registry). Am. J. Cardiol..

[4] Baldus S., Schillinger W., Franzen O., Bekeredjian R., Sievert H., Schofer J., Kuck K.-H., Konorza T., Möllmann H., Hehrlein C. (2012). MitraClip therapy in daily clinical practice: Initial results from the German transcatheter mitral valve interventions (TRAMI) registry. Eur. J. Hear. Fail..

[5] Maisano F., Franzen O., Baldus S., Schäfer U., Hausleiter J., Butter C., Ussia G.P., Sievert H., Richardt G., Widder J.D. (2013). Percutaneous mitral valve interventions in the real world: Early and 1-year results from the ACCESS-EU, a prospective, multicenter, nonrandomized post-approval study of the MitraClip therapy in Europe. J. Am. Coll. Cardiol..

[6] Nickenig G., Estevez-Loureiro R., Franzen O., Tamburino C., Vanderheyden M., Lüscher T.F., Moat N., Price S., Dall’Ara G., Winter R. (2014). Percutaneous mitral valve edge-to-edge repair: In-hospital results and 1-year follow-up of 628 patients of the 2011-2012 Pilot European Sentinel Registry. J. Am. Coll. Cardiol..

[7] Carrasco-Chinchilla F., Arzamendi D., Romero M., de Carlos F.G., Alonso-Briales J.H., Li C.H., Mesa M.D., Arnold R., Frutos A.M.S., Pan M. (2014). Initial experience of percutaneous treatment of mitral regurgitation with MitraClip(R) therapy in Spain. Rev. Esp. Cardiol..

[8] Obadia J.F., Messika-Zeitoun D., Leurent G., Iung B., Bonnet G., Piriou N., Lefèvre T., Piot C., Rouleau F., Carrié D. (2018). Percutaneous Repair or Medical Treatment for Secondary Mitral Regurgitation. N. Engl. J. Med..

[9] Stone G.W., Lindenfeld J., Abraham W.T., Kar S., Lim D.S., Mishell J.M., Whisenant B., Grayburn P.A., Rinaldi M., Kapadia S.R. (2018). Transcatheter Mitral-Valve Repair in Patients with Heart Failure. N. Engl. J. Med..

[10] Kalbacher D., Schäfer U., Bardeleben R.S.V., Eggebrecht H., Sievert H., Nickenig G., Butter C., May A.E., Bekeredjian R., Ouarrak T. (2019). Long-term outcome, survival and predictors of mortality after MitraClip therapy: Results from the German Transcatheter Mitral Valve Interventions (TRAMI) registry. Int. J. Cardiol..

[11] Grasso C., Ince H. (2016). The MitraClip system: Strategies for optimal patient selection and optimised results. EuroIntervention.

[12] Kebler M.J., Whörle J., Rpttbauer W., Markovic S. (2018). Procedural and Clinical Results of the New Mitraclip NT after Percutaneous Edge to Edge Repair of Mitral Valve Regurgitation. Int. J. Cardiovac. Res..

[13] Godino C., Scotti A., Taramasso M., Adamo M., Russo M., Chiarito M., Melillo F., Beneduce A., Pivato C.A., Arrigoni L. (2018). Two-year cardiac mortality after MitraClip treatment of functional mitral regurgitation in ischemic and non-ischemic dilated cardiomyopathy. Int. J. Cardiol..

[14] Keßler M., Seeger J., Muche R., Wöhrle J., Rottbauer W., Markovic S. (2019). Predictors of rehospitalization after percutaneous edge-to-edge mitral valve repair by MitraClip implantation. Eur. J. Heart Fail..

[15] Hamm K., Zacher M., Hautmann M., Gietzen F., Halbfaß P., Kerber S., Diegeler A., Schieffer B., Barth S. (2016). Influence of experience on procedure steps, safety, and functional results in edge to edge mitral valve repair-a single center study. Catheter. Cardiovasc. Interv..

[16] Ledwoch J., Franke J., Baldus S., Schillinger W., Bekeredjian R., Boekstegers P., Hink U., Kuck K.-H., Ouarrak T., Möllmann H. (2014). Impact of the learning curve on outcome after transcatheter mitral valve repair: Results from the German Mitral Valve Registry. Clin. Res. Cardiol..

